# Comparative analysis of gene expression profiles in normal hip human cartilage and cartilage from patients with necrosis of the femoral head

**DOI:** 10.1186/s13075-016-0991-4

**Published:** 2016-05-04

**Authors:** Ruiyu Liu, Qi Liu, Kunzheng Wang, Xiaoqian Dang, Feng Zhang

**Affiliations:** Department of Orthopedics, the Second Affiliated Hospital, Health Science Center, Xi’an Jiaotong University, Xi’an, Shaanxi P.R. China; Key Laboratory of Trace Elements and Endemic Diseases of National Health and Family Planning Commission, School of Public Health, Health Science Center, Xi’an Jiaotong University, Xi’an, Shaanxi P. R. China

**Keywords:** Necrosis of femoral head, Articular cartilage, Gene expression profiles, Gene Ontology

## Abstract

**Background:**

The pathogenesis of necrosis of the femoral head (NFH) remains elusive. Limited studies were conducted to investigate the molecular mechanism of hip articular cartilage damage in NFH. We conducted genome-wide gene expression profiling of hip articular cartilage with NFH.

**Methods:**

Hip articular cartilage specimens were collected from 18 NFH patients and 18 healthy controls. Gene expression profiling of NFH articular cartilage was carried out by Agilent Human 4x44K Gene Expression Microarray chip. Differently expressed genes were identified using the significance analysis of microarrays (SAM) software. Gene Ontology (GO) enrichment analysis of differently expressed genes was performed using the Database for Annotation, Visualization and Integrated Discovery (DAVID). Significantly differently expressed genes in the microarray experiment were selected for quantitative real-time PCR (qRT-PCR) and immunohistochemical validation.

**Results:**

SAM identified 27 differently expressed genes in NFH articular cartilage, functionally involved in extracellular matrix, cytokines, growth factors, cell cycle and apoptosis. The expression patterns of the nine validation genes in qRT-PCR were consistent with that in proteinaceous extracellular matrix (false discovery rate (FDR) = 3.22 × 10^-5^), extracellular matrix (FDR = 5.78 × 10^-5^), extracellular region part (FDR = 1.28 × 10^-4^), collagen (FDR = 3.22 × 10^-4^), extracellular region (FDR = 4.78 × 10^-4^) and platelet-derived growth factor binding (FDR = 5.23 × 10^-4^).

**Conclusions:**

This study identified a set of differently expressed genes, implicated in articular cartilage damage in NFH. Our study results may provide novel insight into the pathogenesis and rationale of therapies for NFH.

**Electronic supplementary material:**

The online version of this article (doi:10.1186/s13075-016-0991-4) contains supplementary material, which is available to authorized users.

## Background

Necrosis of the femoral head (NFH) is a debilitating disease, mainly affecting young adults aged between 35 and 55 years [1]. NFH leads to rapid destruction and dysfunction of the hip joints. About 65–70 % of patients with advanced NFH need total hip replacement [2, 3]. The etiology and pathogenesis of NFH remains elusive, and there is a lack of effective approaches to the prevention and early treatment of NFH.

In the early stages NFH is mainly characterized by the death of osteocytes and bone marrow cells [1, 4]. The reparative reaction of necrotic bone is then initiated. During the repair process the imbalance between osteoclast-mediated bone resorption and osteoblast-mediated bone reformation results in structural damage and collapse of the femoral head. Because osteonecrosis is the representative pathological change in NFH, most studies of NFH have focused on the mechanism of damage to the bone and the bone marrow in the femoral head.

There is significant destruction of the hip articular cartilage during the development of NFH [5, 6]. Degeneration and cracking of the hip articular cartilage increases the instability of hip and accelerates the development of NFH [5, 6]. Prevention and early treatment of hip articular cartilage damage has the potential to slow the development of NFH and relieve hip dysfunction. However, few studies have been conducted to investigate the molecular mechanism of hip articular cartilage damage in NFH. To the best of our knowledge, to date no gene expression profiling of hip articular cartilage has been conducted in NFH, limiting our efforts to clarify the pathogenesis of NFH.

In this study, we conducted genome-wide gene expression profiling of hip articular cartilage in four patients with NFH and four healthy controls. A set of genes differently expressed in hip articular cartilage were identified for NFH. Quantitative real-time PCR (qRT-PCR) was conducted to validate the gene expression profiling results using an independent sample of eight patients with NFH and eight healthy controls. Our results provide novel clues for understanding the molecular mechanism of NFH.

## Methods

### Ethics statement

This study was approved by the Institutional Review Board of Xi’an Jiaotong University. Written informed consent was obtained from all subjects.

### Articular cartilage specimens

**Fig. 1:**
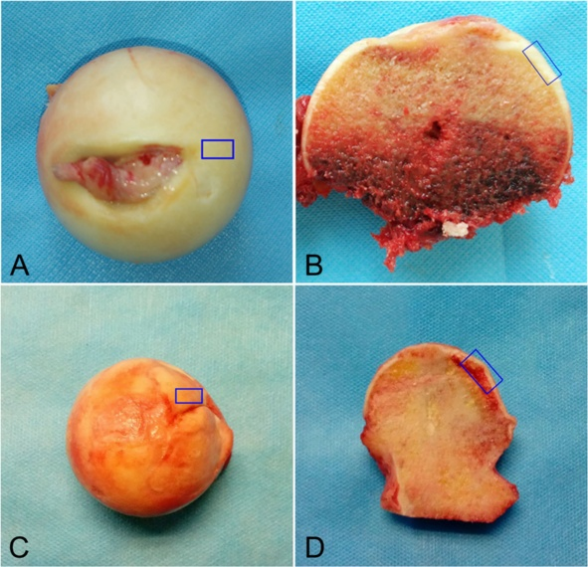
Images of femoral heads from patients (male, 51 years of age) with necrosis of the femoral head (NFH) (**a**, **b**) and healthy controls (male, 53 years of age) (**c**, **d**) in the microarray experiment. *Blue boxes* denote the regions used for collection of specimens from the femoral head

Hip articular cartilage specimens were collected from 18 patients with non-traumatic NFH and 18 healthy control subjects at the Second Affiliated Hospital of Xi’an Jiaotong University. All study subjects were Chinese Han. The NFH patients and control subjects were diagnosed according to clinical manifestations and radiography of the hip assessed by at least two NFH experts [7, 8]. NFH articular cartilage was collected from patients with NFH classified by the the Ficat system as grade III, who were undergoing total hip replacement [7]. Articular cartilage was also obtained from subjects without NFH, who were undergoing total hip replacement within 24 hours of traumatic femoral neck fracture. All cartilage specimens were collected from the antero-superior portions of the femoral head, where the cartilage had collapsed (Fig. 1). Articular cartilage was only used in this study if it had an intact gross appearance and was graded below histological grade 2 [9, 10]. Clinical data for each participant was recorded by doctor-administered questionnaire, including self-reported ethnicity, lifestyle characteristics, health status, and family and medical history. Subjects were excluded if they were identified by assessment of clinical manifestations and radiologic imaging of the hip as having osteoarthritis, rheumatoid arthritis, or other hip disorders. Four, eight, and six NFH-control pairs, matched for age and sex, were used for microarray, qRT-PCR and immunohistochemical analysis, respectively (Table 1).

**Table 1:** Characteristics of study subjects

	NFH		Control	
	Age (years)	Sex	Age (years)	Sex
Microarray	42	Male	45	Male
	41	Male	42	Male
	51	Male	53	Male
	47	Female	47	Female
qRT-PCR	42	Male	42	Male
	42	Male	54	Male
	43	Male	57	Male
	47	Male	61	Male
	47	Male	64	Male
	48	Female	60	Female
	54	Female	61	Female
	57	Female	63	Female

*NFH* necrosis of the femoral head, *qRT-PCR* quantitative real-time PCR

### RNA preparation

The obtained cartilage specimens were rapidly dissected and frozen in liquid nitrogen, and subsequently stored at –80 °C until RNA extraction. Frozen cartilage samples were first rapidly ground in liquid nitrogen using a freezer mill. Total RNA were then isolated from cartilage samples using the Agilent Total RNA Isolation Mini kit (Agilent Technologies, Santa Clara, CA, USA) following the manufacturer’s recommended protocol. The integrity of isolated total RNA was evaluated with 1 % agarose gel electrophoresis. The concentration of isolated total RNA was determined by Agilent ND-1000 (Agilent Technologies) (Additional file 3: Table S1).

### Microarray hybridization

Total RNA was translated into complementary RNA (cRNA) and labeled with Cy3 using the Agilent Quick Amp Labeling kit (Agilent Technologies). Following the Agilent One-Color Microarray-Based Gene Expression Analysis protocol (Agilent Technology), the labeled cRNA was purified using RNeasy Mini Kit (Qiagen, Germantown, MD, USA)). The concentration and specific activity of labeled cRNA were measured by Agilent ND-1000: 1 μg of labeled cRNA was mixed with hybridization buffer and hybridized to the Agilent Human 4x44K Gene Expression Microarray (v2, Agilent Technologies). Hybridization signals were recorded using the Agilent microarray scanner (G2505C), and analyzed by Feature Extraction v11.0 and Agilent GeneSpring GX v12.1 software (Agilent Technologies). The quality of fluorescent spots was evaluated, and the fluorescent spots failing to pass the quality control procedures were excluded for further analysis. Linear and locally weighted scatterplot smoothing (LOWESS) normalization were conducted. The microarray data have been deposited in the Gene Expression Omnibus database [GEO: GSE74089].

### Identification of differently expressed genes

Differently expressed genes were identified using the Significance Analysis of Microarrays (SAM) software, Excel plug-in version 4.01 (http://statweb.stanford.edu/~tibs/SAM/) [11]. To ensure the accuracy of microarray data analysis, the genes presenting both fold changes >3.0 and false discovery rate (FDR) <0.01 were considered as being significantly differentially expressed. The FDR values were calculated by the permutation-based analysis algorithm of SAM [11].

### Gene Ontology enrichment analysis

Gene Ontology (GO) enrichment analysis of differently expressed genes was performed using the functional annotation tool Database for Annotation, Visualization and Integrated Discovery (DAVID) 6.7 (http://david.abcc.ncifcrf.gov/home.jsp) [12]. GO enrichment analysis can integrate the information about disease-related genes and known functional relationships of multiple genes, and can help identify disease-relevant gene sets with known biological functions. In this study significant GO terms were identified at a FDR <0.01.

### Quantitative real-time PCR

qRT-PCR was conducted to validate the accuracy of microarray data using an independent sample of eight patients with NFH and eight healthy controls (Table 1). Based on gene function and results from previous study of joint diseases, nine cartilage development and damage-related differently expressed genes in the microarray experiment were selected for qRT-PCR validation, including ANGPTL4, *ASPN*, *COL1A1*, *COL3A1*, *CRTAC1*, *OGN*, *P4HA2*, *SPP1* and *VKORC1* [13–20]. Glyceraldehyde-3-phosphate dehydrogenase (*GAPDH*) was used as an endogenous invariant control for data normalization. Total RNA was isolated from cartilage specimens, and prepared in the same way as used by the microarray experiment. The isolated total RNA was converted into cDNA using SuperScript III Reverse Transcriptase (Invitrogen, Carlsbad, CA, USA). The ABI Gene Amp PCR System 9700 (Applied Biosystems) was used for cDNA amplification and detection following the manufacturer’s recommended protocol. The expression levels of the nine genes were normalized to the amount of *GAPDH*. Relative fold changes of genes were calculated using the comparative cycle threshold (Ct) equation (2^-△△Ct^): *t* tests were conducted to assess the significance of gene expression differences between articular cartilage in NFH and healthy articular cartilage.

### Immunohistochemical analysis

Hip cartilage specimens were collected from six patients with NFH (three male and three female, age 53.2 ± 5.1 years) and 6 healthy control subjects (three male and three female, age 59.2 ± 4.3 years). The paraformaldehyde-fixed cartilage tissues from patients with NFH and control subjects were rinsed with phosphate-buffered saline (PBS), decalcified and embedded in paraffin. Paraffin-embedded cartilage tissues were sectioned (approximately 5–8 μm thick), and placed on glass slides. For histochemical analysis the cartilage tissue slides were dewaxed in xylene, hydrated with graded ethanol, and stained respectively by hematoxylin and eosin (H&E), toluidine blue (TU) and Safranin O (SO) (Additional file 2: Figure S1). For immunohistochemical analysis, the dewaxed and hydrated cartilage sections were treated with 3 % hydrogen peroxide solution for 10 minutes, rinsed with PBS, and incubated with P4HA2, SPP1 and CRTAC1 antibody (1:50 dilution, Abcam plc, Cambridge, MA, USA) at 4 °C overnight. After washing with PBS, the cartilage sections were incubated with secondary antibody (ZHONGSHAN Golden Bridge Biotechnology, Beijing, China) at 37 °C for 15 minutes, exposed to streptavidin-horseradish peroxidase at 37 °C for 15 minutes, and stained with 3,3-diaminobenzidine (DAB). Four cartilage sections prepared from each cartilage specimen were used for immunohistochemical analysis. In the superficial zone, middle zone and deep zone of the cartilage (Additional file 3: Figure S2), the percentages of positive chondrocytes in 1000 chondrocytes were calculated separately for each cartilage section. Finally, the mean percentage of positive chondrocytes in the four cartilage sections was reported for each cartilage specimen. Significant differences in the expression of the P4HA2, SPP1 and CRTAC1 proteins in cartilage specimens from the six patients with NFH and the six control subjects were assessed using the *t* test. The methods used for the negative control groups were the same as described previously, except that the P4HA2, SPP1 and CRTAC1 antibodies were replaced by PBS.

## Results

### Differently expressed genes in articular cartilage from patients with NFH

SAM identified 24 genes that were significantly upregulated (FDR <0.01) in articular cartilage from patients with NFH (Table 2). The biological function of the 24 upregulated genes mainly includes extracellular matrix (11 genes), cytokines (3 genes), growth factors (2 genes), cell cycle (2 genes) and apoptosis (1 gene). The average gene expression ratio of the 24 upregulated genes was 16.77. Additionally, SAM identified three significantly downregulated genes in articular cartilage from patients with NFH, including *TMEM171* (FDR = 5.61 × 10^-5^), *MDK* (FDR = 4.38 × 10^-4^) and *VKORC1* (FDR = 4.02 × 10^-3^). The average gene expression ratio of the three downregulated genes was 0.42.

**Table 2:** Differently expressed genes in articular cartilage from patients with necrosis of the femoral head

Gene	Genbank ID	Function	Ratio
*COL5A1*	NM_000093	Extracellular matrix	17.32 ± 4.95
*CRTAC1*	NM_018058	Extracellular matrix	22.67 ± 3.19
*CRLF1*	NM_004750	Cytokines	62.28 ± 6.61
*COL6A3*	NM_004369	Extracellular matrix	12.80 ± 3.25
*COL3A1*	NM_000090	Extracellular matrix	13.56 ± 4.24
*OGN*	NM_033014	Extracellular matrix	10.78 ± 3.63
*MT1F*	NM_005949	Metallothionein	6.08 ± 0.78
*ANGPTL4*	NM_139314	Growth factor	26.83 ± 2.65
*IGFBP7*	NM_001553	Growth factor	22.89 ± 6.02
*COL6A1*	NM_001848	Extracellular matrix	8.73 ± 2.91
*CRIP1*	NM_001311	Cell cycle	7.79 ± 1.88
*SPP1*	NM_001040058	Extracellular matrix	17.61 ± 4.10
*ASPN*	NM_017680	Extracellular matrix	22.15 ± 5.28
*MXRA7*	NM_001008529	Extracellular matrix	7.94 ± 2.21
*NFIL3*	NM_005384	Transcription	11.05 ± 1.92
*MINOS1-NBL1*	NM_001204088	Miscellaneous	5.76 ± 1.57
*METRNL*	NM_001004431	Miscellaneous	11.77 ± 3.40
*P4HA2*	NM_004199	Extracellular matrix	6.96 ± 1.47
*COL1A1*	NM_000088	Extracellular matrix	51.81 ± 15.49
*TSC22D3*	NM_004089	Cytokines	7.39 ± 1.92
*ID2*	NM_002166	Cell cycle	20.62 ± 5.78
*PRG4*	NM_005807	Cytokines	13.87 ± 3.72
*CD55*	NM_000574	Apoptosis	6.53 ± 1.52
*STEAP1*	NM_012449	Transmembrane protein	7.31 ± 2.21
*TMEM171*	NM_173490	Miscellaneous	0.34 ± 0.05
*MDK*	NM_001012334	Cytokines	0.47 ± 0.02
*VKORC1*	AK125618	Miscellaneous	0.44 ± 0.09

### GO enrichment analysis

GO enrichment analysis was performed to investigate the molecular mechanism of differently expressed genes involved in damage to the articular cartilage in NFH. We detected six GO terms significantly enriched in the differently expressed genes in articular cartilage from patients withg NFH (Table 3). They are proteinaceous extracellular matrix (FDR = 3.22 × 10 ^-5^), extracellular matrix (FDR = 3.22 × 10^-5^), extracellular region part (FDR = 1.28 × 10^-4^), collagen (FDR = 3.22 × 10^-4^), extracellular region (FDR = 4.78 × 10^-4^) and platelet-derived growth factor binding (FDR = 5.23 × 10^-4^).

**Table 3:** Gene Ontology enrichment analysis results for differently expressed genes

GO term	GO ID	FES^a^	FDR
Proteinaceous extracellular matrix	GO:0005578	15.63	3.22 × 10^-5^
Extracellular matrix	GO:0031012	14.50	5.78 × 10^-5^
Extracellular region part	GO:0044421	6.95	1.28 × 10^-4^
Collagen	GO:0005581	79.39	3.22 × 10^-4^
Extracellular region	GO:0005576	4.15	4.78 × 10^-4^
Platelet-derived growth factor binding	GO:0048407	224.81	5.23 × 10^-4^

^a^Fold enrichment score calculated using the Database for Annotation, Visualization and Integrated Discovery (DAVID)

### qRT-PCR validation

**Fig. 2:**
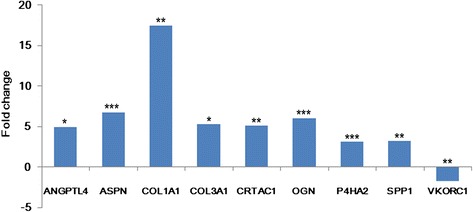
Results of quantitative real-time PCR. *P values <0.05; ^†^ *P* values <0.01; ^‡^ *P* values <0.001, calculated by the *t* test

Nine significantly differently expressed genes in the microarray experiment were selected for qRT-PCR using an independent sample of eight patients with NFH and eight healthy controls (Fig. 2). The expression patterns of the nine validation genes in qRT-PCR were consistent with that in the microarray experiment, including *ANGPTL4* (ratio = 4.89, *P* = 0.05), *ASPN* (ratio = 6.69, *P* value = 3.90 × 10^-5^), *COL1A1* (ratio = 17.43, *P* value = 0.01), *COL3A1* (ratio = 5.33, *P* value = 0.02), *CRTAC1* (ratio = 5.08, *P* value = 4.24 × 10^-3^), *OGN* (ratio = 5.99, *P* value = 9.14 × 10^-4^), *P4HA2* (ratio = 3.12, *P* value = 1.30 × 10^-5^), *SPP*1 (ratio = 3.20, *P* value = 2.14 × 10^-3^), and *VKORC1* (ratio = 0.56, *P* value = 1.76 × 10^-3^).

### Immunohistochemical analysis

**Fig. 3:**
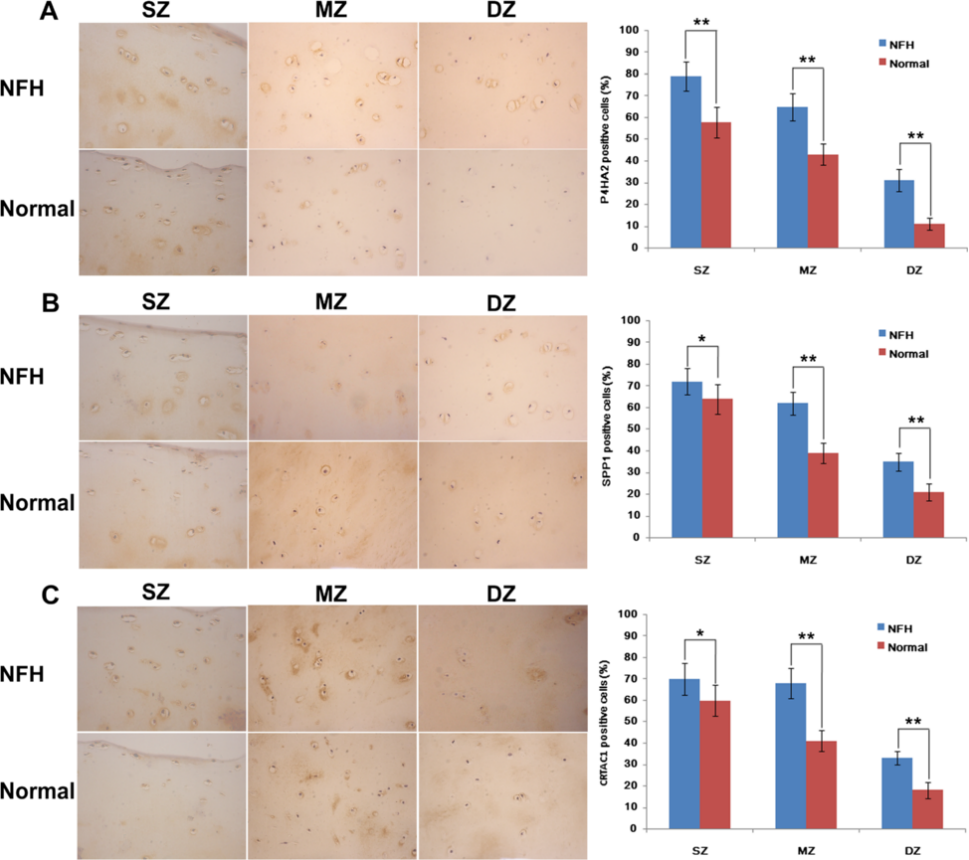
Immunohistochemistry results for P4HA2 (**a**), SPP1 (**b**) and CRTAC1 (**c**) proteins in cartilage from patients with necrosis of the femoral head (*NFH*) and normal hip cartilage. Original magnification × 400 of the superficial zone (*SZ*), middle zone (*MZ*) and deep zone (*DZ*). The expression of the P4HA2, SPP1 and CRTAC1 proteins in cartilage from patients with NFH was significantly higher than in normal hip cartilage in the SZ, MZ and DZ: n = 6 in each group. *P values <0.05; &*P* values <0.001

Immunohistochemical experiments were performed to evaluate the expression levels of the P4HA2, SPP1 and CRTAC1 proteins in NFH and normal cartilage. As shown in Fig. 3, expression of the P4HA2, SPP1 and CRTAC1 proteins in the superficial zone, middle zone, and deep zone of cartilage from patients with NFH was significantly higher than in normal hip cartilage (all *P* values <0.05). Additionally, we also observed decreased expression levels of the P4HA2, SPP1 and CRTAC1 proteins in the superficial zone, and middle zone to deep zone of cartilage from both patients with NFH and normal controls.

## Discussion

Previous studies have implicated degeneration and cracking of hip articular cartilage in the development of NFH [5, 6]. We compared the gene expression profiles of articular cartilage from patients with NFH in articular cartilage from subjects without NFH, to try and understand the mechanism of damage to the articular cartilage in NFH. We identified 24 upregulated genes and 3 downregulated genes in articular cartilage in NFH. The 27 differently expressed genes are functionally involved mainly in the extracellular matrix, cytokines, growth factors, cell cycle and apoptosis. To the best of our knowledge, this is the first gene expression profile study of articular cartilage in NFH. Our study results may provide novel insight into the pathogenesis of NFH and a rationale for therapies.

We found that 11 extracellular-matrix-related genes were significantly upregulated in NFH, including *ASPN*, *COL1A1*, *COL3A1*, *COL5A1*, *COL6A1*, *CRTAC1*, *MXRA7*, *OGN*, *P4HA2*, and *SPP1*: 4 of these are collagen genes. *COL1A1* encodes pro-alpha1 chains of type I collagen, which is abundant in bone. *COL1A1* mutations are one of the major causes of osteogenesis imperfecta [21]. *COL3A1* encodes the pro-alpha1 chains of type III collagen, which is widely expressed in the vascular system. Loeser et al. observed significant upregulation of *COL3A1* in an osteoarthritis mice model [22]. *COL5A1* encodes the alpha chain of type V collagen, which is a minor component of connective tissue. In an animal study dysfunction of *COL5A1* was found to generate an abnormal joint phenotype, such as joint laxity and early-onset osteoarthritis [23]. *COL6A1* encodes the alpha 1 subunit of type VI collagen, which is a major structural component of microfibrils. *COL6A1* mutations have been linked to Bethlem myopathy with joint contractures [24]. *P4HA2* encodes procollagen-proline, 2-oxoglutarate 4-dioxygenase, which is a key collagen synthesis enzyme [25]. *P4HA2* knock-out mice have defects in skeletal growth and development [26]. In patients with NFH the upregulated expression of collagen and the collagen synthesis enzyme may be explained by enhanced repairing activity in articular cartilage defects with fibrous tissue. *ASPN* encodes cartilage extracellular protein asporin, which is able to negatively regulate the chondrogenesis of articular cartilage through blocking transforming growth factor (TGF)-beta/receptor interaction in chondrocytes [27]. It has also been found to be involved in damage to the articular cartilage in osteoarthritis and rheumatoid arthritis [13, 28–30]. *SPP1* encodes secreted phosphoprotein 1 (also named osteopontin), which is implicated in the attachment of osteoclasts to mineralized bone matrix. The association between SPP1 and osteoarthritis has been demonstrated [31]. SPP1-deficient mice exhibit accelerated development of osteoarthritis [32]. Yamamoto et al. found that SPP1 contributes to osteoclast-mediated bone resorption and joint inflammatory responses in the mouse model of rheumatoid arthritis [33]. *OGN* encodes osteoglycin, which is capable of inducing ectopic bone formation and regulating cardiovascular development [34, 35]. *CRTAC1* encodes cartilage acidic protein 1, which is expressed in the deep zone in articular cartilage. CRTAC1 acts as a biomarker for distinguishing chondrocytes from osteoblasts and mesenchymal stem cells [17].

With respect to cytokines and growth factors, we observed significant upregulation of the *PRG4* and *ANGPTL4* genes in articular cartilage from patients with NFH. *PRG4* encodes proteoglycan 4, which acts as a boundary lubricant at the surface of articular cartilage [36, 37]. Using transgenic mice and intra-articular adenoviral virus gene transfer, Ruan et al. demonstrated that PRG4 protected against the development of osteoarthritis in mice [38]. The *ANGPTL4* gene encodes angiopoietin-like 4, which is an important regulator of angiogenesis [39]. Perdiguero observed that ANGPTL4-deficient mice have impaired angiogenesis and increased vascular leakage [40]. ANGPTL4 also simulates endothelial cell growth and tubule formation, and prevents endothelial cell apoptosis [39, 41]. The role of ANGPTL4 in the dysfunctional blood supply in NFH is worthy of further study.

## Conclusion

We conducted a gene expression profile study of the articular cartilage in NFH. We identified a set of differently expressed genes, implicated in the destruction of articular cartilage in NFH. Further biological studies are warranted to confirm our findings and clarify the potential mechanism of the identified genes involved in the development of NFH.

## Additional files

Additional file 1: Table S1. RNA concentrations of study samples. (DOCX 27 kb) Additional file 2: Figure S1. Hematoxylin and eosin (HE), safranin O (SO) and toluidine blue (TU) staining of NFH articular cartilage and normal articular cartilage. (TIF 10334 kb) Additional file 3: Figure S2. Cartilage zones for immunohistochemical analysis of P4HA2 (A), SPP1 (B) and CRTAC1 (C) proteins. The arrows indicate respectively the superficial zone (SZ), middle zone (MZ) and deep zone (DZ) of articular cartilage. (TIF 13919 kb)

## Abbreviations

cRNA: complementary RNA
DAVID: Database for Annotation, Visualization and Integrated Discovery
FDR: false discovery rate
GAPDH: glyceraldehyde-3-phosphate dehydrogenase
GO: Gene Ontology
H&E: hematoxylin and eosin
NFH: necrosis of femoral head
PBS: phosphate-buffered saline
qRT-PCR: quantitative real-time PCR
SAM: significance analysis of microarrays
