# Supplementary material for: Comparative analysis of gene expression profiles in normal hip human cartilage and cartilage from patients with necrosis of the femoral head
Source: Arthritis Res Ther. 2016 May 4;18:98. doi: 10.1186/s13075-016-0991-4 (PMC4857375; doi:10.1186/s13075-016-0991-4)
Supplement: Additional file 1: Table S1. — RNA concentrations of study samples. (DOCX 27 kb) [file 13075_2016_991_MOESM1_ESM.docx]

**Table S1.** RNA concentrations of study samples

|  | **NFH^a^** | |  | **Control** | |
| --- | --- | --- | --- | --- | --- |
|  | Age (years) | Concentrations(ng/μl) |  | Age (years) | Concentrations(ng/μl) |
| **Microarray** | 42 | 154.79 |  | 45 | 167.87 |
|  | 41 | 223.58 |  | 42 | 105.14 |
|  | 51 | 145.59 |  | 53 | 141.51 |
|  | 47 | 189.74 |  | 47 | 140.03 |
| **qRT-PCR** | 42 | 154.79 |  | 42 | 167.87 |
|  | 42 | 90.09 |  | 54 | 141.51 |
|  | 43 | 223.58 |  | 57 | 105.14 |
|  | 47 | 189.74 |  | 61 | 149.78 |
|  | 47 | 113.40 |  | 64 | 140.92 |
|  | 48 | 360.31 |  | 60 | 169.89 |
|  | 54 | 145.59 |  | 61 | 162.44 |
|  | 57 | 148.78 |  | 63 | 140.03 |

^a^ necrosis of femoral head.
