# Supplementary figures and images for: Comparative analysis of gene expression profiles in normal hip human cartilage and cartilage from patients with necrosis of the femoral head
Source: Arthritis Res Ther. 2016 May 4;18:98. doi: 10.1186/s13075-016-0991-4 (PMC4857375; doi:10.1186/s13075-016-0991-4)

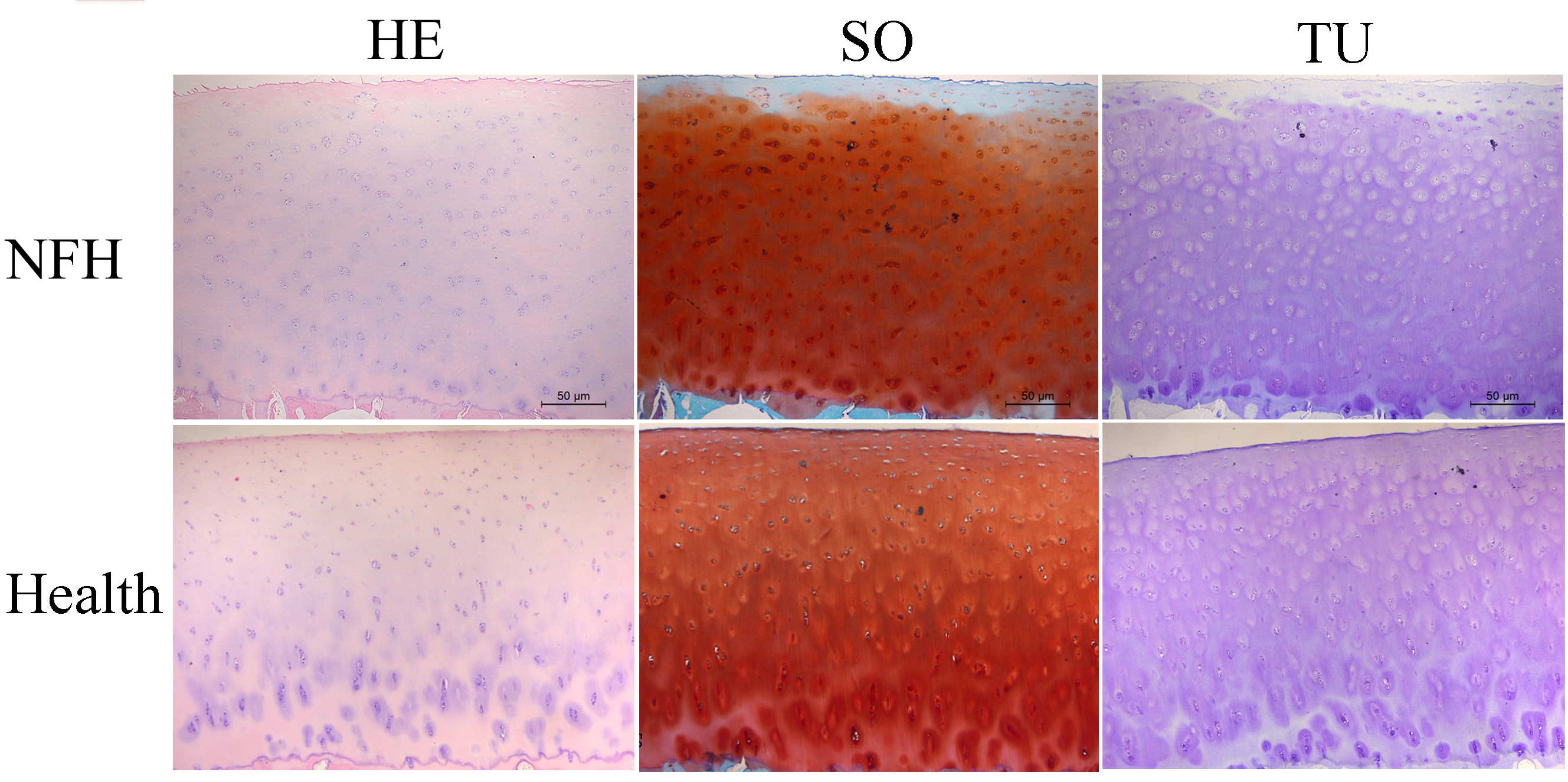

Supplement: Additional file 2: Figure S1. — Hematoxylin and eosin (HE), safranin O (SO) and toluidine blue (TU) staining of NFH articular cartilage and normal articular cartilage. (TIF 10334 kb) [file 13075_2016_991_MOESM2_ESM.tif]

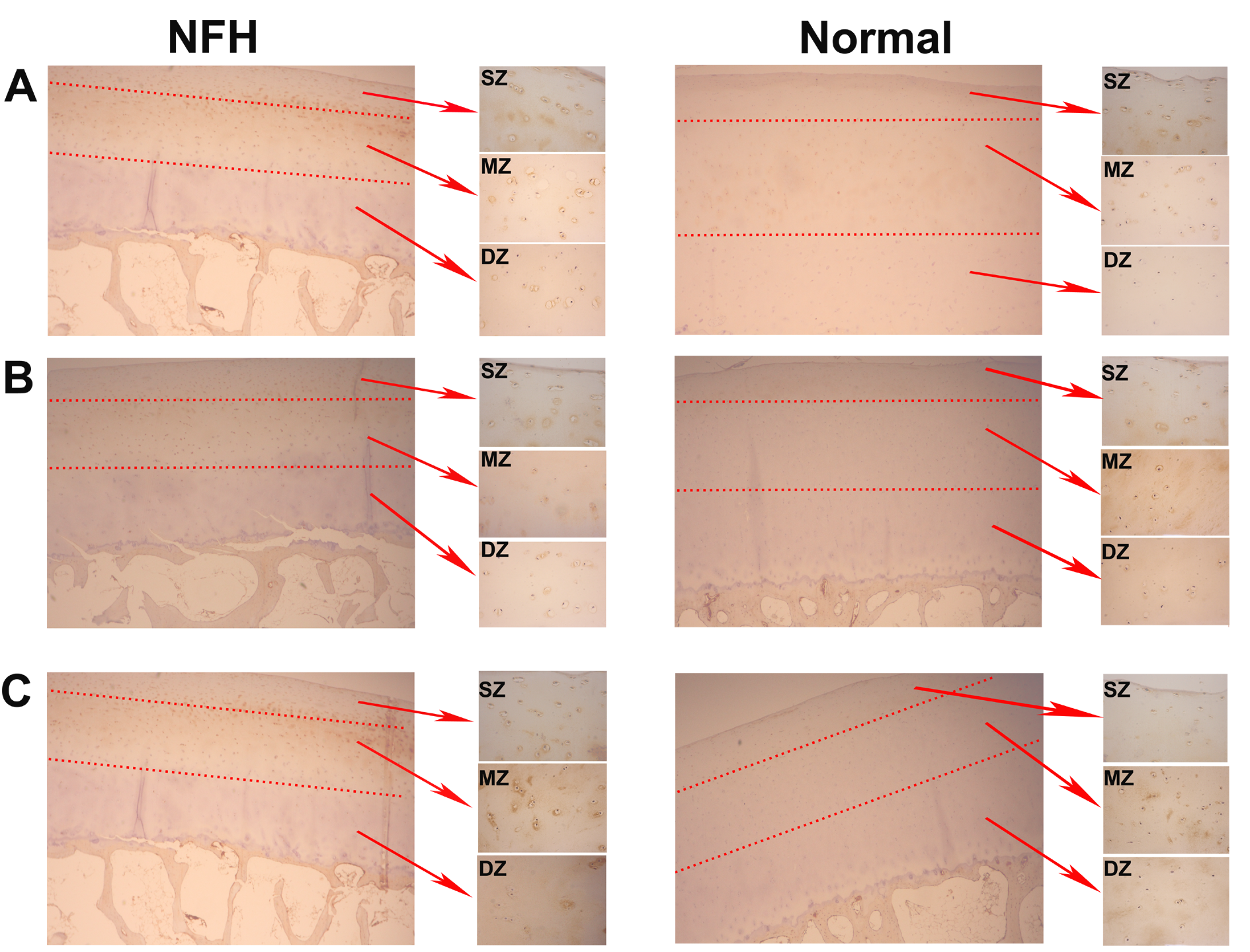

Supplement: Additional file 3: Figure S2. — Cartilage zones for immunohistochemical analysis of P4HA2 (A), SPP1 (B) and CRTAC1 (C) proteins. The arrows indicate respectively the superficial zone (SZ), middle zone (MZ) and deep zone (DZ) of articular cartilage. (TIF 13919 kb) [file 13075_2016_991_MOESM3_ESM.tif]
